# Effects of transcutaneous electrical nerve stimulation on pain, function, and descending inhibition in people with non-specific chronic low-back pain: a study protocol for a randomized crossover trial

**DOI:** 10.1186/s13063-024-08089-7

**Published:** 2024-04-06

**Authors:** Richard E. Liebano, Kathleen A. Sluka, Joshua Roy, Meghan Savinelli, Dana L. Dailey, Sean P. Riley

**Affiliations:** 1 Department of Rehabilitation Sciences, University of Hartford, 200 Bloomfield Avenue, West Hartford, CT 06117 USA; 2 Department of Physical Therapy and Rehabilitation Science, 1-242 MEB, University of Iowa, Iowa City, IA 52242 USA; 3 Department of Physical Therapy and Rehabilitation Science, Roy J and Lucille A Carver College of Medicine, University of Iowa, Iowa City, IA 52242 USA; 4 Department of Physical Therapy, St. Ambrose University, Davenport, IA 52803 USA; 5 Hartford Healthcare Rehabilitation Network, 330 Western Blvd #101, Glastonbury, CT, 06033 USA

**Keywords:** Low back pain, Transcutaneous electric nerve stimulation, Study protocol, Randomized controlled trial, Nociception, Pain measurement, Pain threshold, Analgesics

## Abstract

**Background:**

Low back pain (LBP) is a significant public health problem, is very prevalent, and is often characterized by the persistence of symptoms. Transcutaneous electrical nerve stimulation (TENS) may benefit people with chronic LBP because it can activate descending inhibitory pathways and inhibit central excitability. However, previous studies that have investigated the effects of TENS on pain in people with LBP have failed to use proper intensities of current, and the timing of the assessment of pain was not performed during the peak of the analgesic response or functional activities. Therefore, the present study aims to assess the effects of TENS on measures of pain, function, and descending inhibition using the maximal tolerable intensity of TENS in participants with LBP.

**Methods/design:**

This study will be a randomized crossover trial. The participants for this study will be recruited from various places, including the University of Hartford, physical therapy clinics, and local businesses in the Hartford area, as well as online websites geared towards clinical trial recruitment. A total of 34 participants will receive all three treatments: active TENS, placebo TENS, and no treatment control. The treatment order will be randomized using a website-based randomization tool. For active TENS, a modulating frequency of 2–125 Hz will be applied with a variable pulse duration and maximal tolerable intensity for 30 min. The TENS will be left on for post-treatment testing to assess the effects during its maximally effective period for a total of 50 to 60 min. Furthermore, the intensity may be turned down if muscle twitching is present to ensure blinding of the evaluator. For placebo TENS, the unit will deliver current for 45 s, ramping to 0 in the last 15 s. The primary outcome will be pain intensity at rest and with movement, determined using the numerical pain rating scale. The secondary outcomes will be pressure pain threshold, heat pain threshold, temporal summation of pain, conditioned pain modulation, sit-to-stand test, and repeated trunk flexion. The assessments will be performed immediately before and after treatment. Statistical analysis of the data obtained will consider a significance level of *p* < 0.05.

**Discussion:**

This study will provide evidence concerning the effects and mechanisms of TENS treatment in participants with chronic non-specific low back pain. The outcomes, including pain, function, and descending inhibition, will help us gain a greater understanding of how TENS can be used for these participants.

**Trial registration:**

ClinicalTrials.gov NCT05812885. Registered on 24th May 2023.

## Introduction

### Background and rationale {6a}

Low back pain (LBP) is a significant public health problem [1], is very prevalent [2–4], and is often characterized by the persistence of symptoms [2]. Unfortunately, substantial improvements in people with chronic LBP are rare, causing most people to live with the pain [2]. People with chronic LBP may have an exaggerated pain response to nociceptive input that may also cause symptoms distant from the site of the primary symptoms [5]. Historically, these symptoms were thought to be related to pathoanatomic changes to the muscles, ligaments, or joints. The severity of these structural changes weakly correlates with the clinical presentation and, in most cases, is not directly related to diagnostic image findings [6]. Individuals with chronic musculoskeletal pain generally show signs of central excitability. The central excitability may be directly correlated with the intensity and duration of pain [7]. Therefore, it is essential to reduce pain intensity and minimize the duration of pain to reduce central excitability [8].

Pain relief for individuals with chronic LBP should be aimed at treatments that reduce central excitability and increase central inhibition [8]. Research into chronic LBP treatment has demonstrated strong evidence that different types of exercise decrease pain and improve quality of life [9]. However, exercise itself may be painful, which may prevent a person from exercising. Thus, treatments aimed at decreasing pain should improve a person’s ability to exercise and participate in activities of daily living.

One treatment aimed at reducing central excitability and increasing central inhibition is transcutaneous electrical nerve stimulation [10, 11]. TENS is a “non-pharmacological” treatment for pain that is inexpensive, safe, and easy to use. Prior studies show that TENS utilizes opioid receptors both spinally and supraspinally to reduce sensitization of dorsal horn neurons, excitatory neurotransmitter release, and hyperalgesia [12]. Thus, TENS may be particularly useful in people with chronic LBP because it can activate descending inhibitory pathways (reduced in chronic low back pain patients) and inhibit central excitability (increased in chronic LBP patients).

Previous studies that have investigated the effects of TENS on pain in LBP patients have failed to use proper intensities of current, and the timing of the assessment of pain was not performed during the peak of the analgesic response or during functional activities [13]. Previous studies show that inadequate intensities do not reduce pain or increase pressure pain thresholds [12]. Further, TENS typically does not affect resting pain after abdominal surgery, while changes in pressure pain thresholds and pain with movement are reduced [10, 14, 15]. A recent randomized controlled trial addressed these limitations and showed a significant reduction in movement-evoked pain and fatigue with TENS in individuals with fibromyalgia [11]. However, it is unclear if the same reduction, when using adequate experimental design, will also reduce pain in individuals with low back pain. Thus, the current study proposes to assess the effects of TENS on measures of pain, function, and descending inhibition using the maximal tolerable intensity of TENS applied to a large area of the trunk in people with non-specific chronic low-back pain.

### Objectives {7}

This study aims to assess the effects of TENS on measures of pain, function, and descending inhibition in people with chronic non-specific LBP.

### Trial design {8}

The study will be a crossover design and will be conducted at the University of Hartford. The study protocol was approved by the Institutional Review Board (IRB) of the University of Hartford, it was registered online on ClinicalTrials.gov, and will be reported according to CONSORT guidelines [16]. The protocol was developed following the recommendations of SPIRIT (Standard Protocol Items: Recommendations for Interventional Trials). The RCT will have a blinded evaluator.

## Methods: participants, interventions, and outcomes

### Study setting {9}

The study will be carried out at the Department of Rehabilitation Sciences of the University of Hartford, West Hartford, CT, USA.

### Eligibility criteria {10}

To determine eligibility for participation in the study, potential candidates must fill out an online form. The form will collect information about their prior use of TENS, any changes in back and leg sensitivity, pregnancy status, history of chronic illness, presence of pacemaker, epilepsy, skin allergies, injuries or diseases, willingness to change pain medication within the next 3 weeks, peripheral neuropathies, and history of serious injury, illness, or surgery on their back or lower limbs. Once the screening form is completed, a research team member will contact the candidates via phone to ensure they meet all the inclusion and exclusion criteria.

#### Inclusion criteria

- Individuals with non-specific LBP
- On a stable medication regimen during the 4 weeks preceding the study
- Chronic LBP for at least 3 months
- Numerical pain rating scale (NPRS) score ≥  3 and ≤ 8 for pain intensity
- Age between 18 and 60 years [17, 18]
- Men and women
- TENS naive or have not used TENS for 5 years

#### Exclusion criteria

- Serious spinal disorders, such as fractures, tumors, or inflammatory arthritis disease; nerve root disorders confirmed by neurological tests
- Neurological or rheumatological diseases
- Fibromyalgia or other chronic pain condition
- Severe cardiorespiratory disease
- Pregnancy
- Epilepsy
- Skin infection, lesions, or change in sensation at the TENS application site
- Cancer
- Cardiac pacemaker, defibrillator or implanted stimulator
- Skin allergy to electrodes
- Use of opioids
- Individuals who have ingested any sedative, anti-inflammatory or analgesic medications, and alcoholic substance in the last 48 h before the intervention.

### Who will take informed consent? {26a}

Data collection will only start after the participants have signed an informed consent form, which the evaluator will explain.

### Additional consent provisions for collection and use of participant data and biological specimens {26b}

N/A. No biological specimens will be collected as part of this trial.

## Interventions

### Explanation for the choice of comparators {6b}

Previous studies regarding TENS application in participants with chronic LBP have failed to measure pain during the peak of the analgesic effect or have not used adequate current intensities. Therefore, the proposed research will assess the effects of TENS immediately after the intervention and use the maximal tolerable intensity.

### Intervention description {11a}

#### Electrode placement

Four 5  ×  5-cm electrodes (ValuTrode®; Axelgaard, Fallbrook, CA) will be placed over the lumbar paraspinal muscles, immediately above and below the spinal level corresponding to pain complaint, for treatment [13, 19]. These self-adhesive electrodes are commercially available and will be purchased for each subject.

#### Active TENS

An EMPI Select TENS unit will be used to deliver the active TENS intervention. A modulating frequency TENS (2–125 Hz) will be applied with a variable pulse duration, waveform of an asymmetrical biphasic square wave, and maximal tolerable intensity [11, 15]. With this specific unit, current intensity ranges from 0.5 to 60.0 mA and the participants will be educated prior to intervention on what they will feel. TENS will be placed for 30 min after baseline testing and then remain on for the post-assessment tests to assess the effects during its maximally effective period [10] for a total of 50 to 60 min. Every 5 min, the subject will be asked if the intensity can be increased, decreased or remain the same to maintain the same level of intensity. The intensity may be turned down if muscle twitching is present during post-assessment testing maintain blinding of the evaluator.

#### Placebo TENS

An EMPI Select TENS unit will be used to deliver the placebo TENS intervention. The placebo TENS unit appears identical to that of the active TENS unit and will deliver current for 45 s, ramping to 0 in the last 15 s. This allows the person receiving the TENS to be blinded to the treatment applied.

#### No treatment control

Participants will receive no TENS treatment but will perform a quiet rest period of 30 min instead of TENS. All other procedures will be the same. Therefore, this group will control for the placebo effect and repeated testing.

### Criteria for discontinuing or modifying allocated interventions {11b}

There will be no changes in the treatment allocation order. If participants discontinue treatment, recent data will be computed for the analyses according to the intention-to-treat principle, and the reason for the withdrawal from the study will be recorded.

### Strategies to improve adherence to interventions {11c}

To minimize data loss, all participants will be guided when they sign the informed consent form and commit to attending on the scheduled treatment dates. Participants will receive an appointment card for scheduled sessions. An evaluator will be responsible for notifying and monitoring the participants weekly (via telephone contact, text message, and/or email) and accompanying them during the research. In cases of abandonment or impossibility of continuing the study, the data will be analyzed according to an intention-to-treat protocol.

### Relevant concomitant care permitted or prohibited during the trial {11d}

During the experiment, concurrent treatments for chronic diseases such as diabetes and hypertension will be accepted. Throughout the trial, participants will be asked not to start any other interventions or pain medications as it may influence outcomes.

### Provisions for post-trial care {30}

The interventions are designed to inflict minimal to no harm, and no compensation for harm is deemed necessary.

### Outcomes {12}

The primary outcome will be pain intensity at rest and with movement, determined using the NPRS. The secondary outcomes will be pressure pain threshold (PPT), heat pain threshold (HPT), temporal summation (TS) of pain, conditioned pain modulation (CPM), sit-to-stand test, and repeated trunk flexion. CPM will be used to assess descending inhibition. Sit-to-stand and repeated trunk flexion will be used to assess function. These outcomes will be measured twice, once as a baseline testing, and then again post-treatment.

#### Numeric pain rating scale (NPRS)

Resting pain intensity and pain with movement (during the sit-to-stand and repeated flexion tests) at the lumbar spine will be assessed before and after intervention (primary outcome measures). We will be using an 11 point NPRS for pain intensity measurements. Pain scores range from 0 representing “no pain” and 10 representing the “worst pain imaginable” [20].

#### Sit-to-stand test

The sit-to-stand test is a test of strength for the lower body. Five sit-to-stand repetitions will be completed in two trials, with an average of two trials for scoring [21]. The time it takes to complete five repetitions as quickly as possible will be recorded. This test has good reliability (ICC > 0.95) and validity (*r* = 0.59 to 0.88) [21]. The test will be conducted before and after the treatment session to assess a change in speed of sitting to standing, representing a functional change concerning lower extremity strength in response to TENS. The participants will be asked to rate pain at the end of test.

#### Repeated trunk flexion

From a neutral standing position, the subject will be required to flex the trunk to the limit of the trunk flexion range of motion and return to the upright position as fast as tolerable. This activity will be repeated ten times, and the total procedure will be timed with a stopwatch [22, 23]. The task will be repeated after a 30-s pause, and the average time of the two tasks will be the resulting score [21, 23]. This test has good reliability (ICC 0.89) [22]. The participants will rate pain at the end of test.

#### Pressure pain threshold (PPT)

A digital pressure algometer will measure the pain threshold to deep mechanical stimuli. The AlgoMed Computerized Pressure Algometer from the manufacturer Medoc will be utilized. This digital pressure pain algometer is a hand-held, software-based device that provides real-time visual and auditory feedback. The software provides a graphic display of the test displaying the applied pressure rate, to help ensure a constant rate is being applied. The device also provides an auditory cue for when the button has been activated. A 1-cm^2^ algometer probe will apply pressure at 40 kPa/s. Participants will be instructed to activate a button when the sensation of pressure becomes painful, and this value will be recorded. This method will register mean pressure pain thresholds in the lumbar region, forearm, and leg. The intra-rater reliability of the measurement of PPT will be performed in 10 asymptomatic subjects by a single evaluator at 48-h intervals. Reliability will be estimated by calculating the intraclass correlation coefficients (ICCs).

Measures will be done over 2 points marked bilaterally with a permanent marker, the first located 5 cm lateral to the L3 spinous process [24] and the second 5 cm lateral to the L5 spinous process [25]. In addition, a point will also be marked over the anterior tibialis muscle of the right and left leg 5 cm from the tibial tuberosity [26, 27]. There will also be a point marked and measured on the right and left extensor carpi radialis muscle, 5 cm distal from the elbow crease.

#### Heat pain threshold (HPT)

Superficial heat pain sensitivity will be assessed using a handheld thermode (Medoc TSA-II, Israel) with a single 30 × 30-mm probe placed in the midline for each lumbar segment (L5 and L3) centered at the spinous process ensuring complete contact between skin surface and probe. The baseline temperature will be pre-set to 32°C. During testing, the temperature will increase at a rate of 1 °C/s until the participant reports the temperature as painful by pressing an indicator button. Maximum temperature will be pre-set at 50 °C; if no pain had been elicited by then, this will be recorded as the heat pain threshold [28]. Using the thermode to indicate the HPT has excellent intra-rater reliability (ICC 0.86 to 0.93) when tested at the spine of asymptomatic volunteers [29].

The PPT and HPT recordings will be performed, with the patient in the seated position, at each spinal segment three times. The segments will be tested in a pre-determined, computer-generated, random order with 10-s rest intervals between each test. In addition, before data collection, two test trials of each pain threshold assessment will be performed at the dominant forearm to familiarize participants with the procedure [28].

#### Temporal summation (TS)

TS will be induced by a pressure algometer. The intra-rater reliability of pain TS will be tested in 10 asymptomatic participants by a single evaluator at 48-h intervals. Reliability will be estimated by calculating the ICCs. The area selected for TS analysis will be the site indicated as the lower pain threshold in the low back algometry. Ten stimuli will be performed using the algometer on the selected region. Each TS stimulus will be maintained for 1 s before being released, and the stimuli will be spaced at 1-s intervals. Participants will be instructed to report pain using the NRS at the first, fifth, and tenth stimulus [30]. The TS assessment will be performed before and after TENS application.

#### Conditioned pain modulation

A cold pressor test will be used to assess the activation of the conditioned pain modulation [31]. An ARTIC A25 Refrigerated Circulator (Thermo Scientific, Newington, NH, USA) will be the instrument used to administer the cold pressor test. The cold pressor test is as follows: The subject will have PPT on one point of their low back before immersion. Then, CPM will be induced by immersing the participants’ dominant hand up to the wrist into a 5 °C refrigerated circulating water bath. After 20 s, they will then be asked to rate the pain of their dominant hand as it is still immersed on a 0 to 10 scale. The PPT at the low-back algometry point will be recorded 30 s after immersion while the participants hand remains immersed. The magnitude of CPM will be assessed by calculating the PPT variation from pre-immersion values, where positive values represent hypoalgesia and negative values represent hyperalgesia.

#### Study blinding assessment

To assess the blinding of the outcomes assessor, the assessor will be asked, “What treatment did the subject receive today?” The choices for the outcome assessor will be: active TENS, placebo TENS or no TENS. The responses to these questions will be recorded and compared to assess the blinding of the outcomes assessor.

### Participant timeline {13}

Participants will be asked to complete a self-administered questionnaire that collects demographic information such as age, sex, marital status, education level, ethnicity, race, income, and history of surgeries. Weight will be measured using a calibrated electronic scale, height will be measured using a stadiometer, and the body mass index will be calculated. Participants will be randomized to treatment group order after providing written consent. The order of active TENS, placebo TENS and no TENS will be randomized via the website (randomization.com). Simple randomization results will be concealed in sealed opaque envelopes with consecutive numbers and will not be available to the outcomes assessor. The envelopes will be signed, dated, and opened by the TENS allocator before the TENS application and after the outcomes assessor has left the room.

**Fig. 1:**
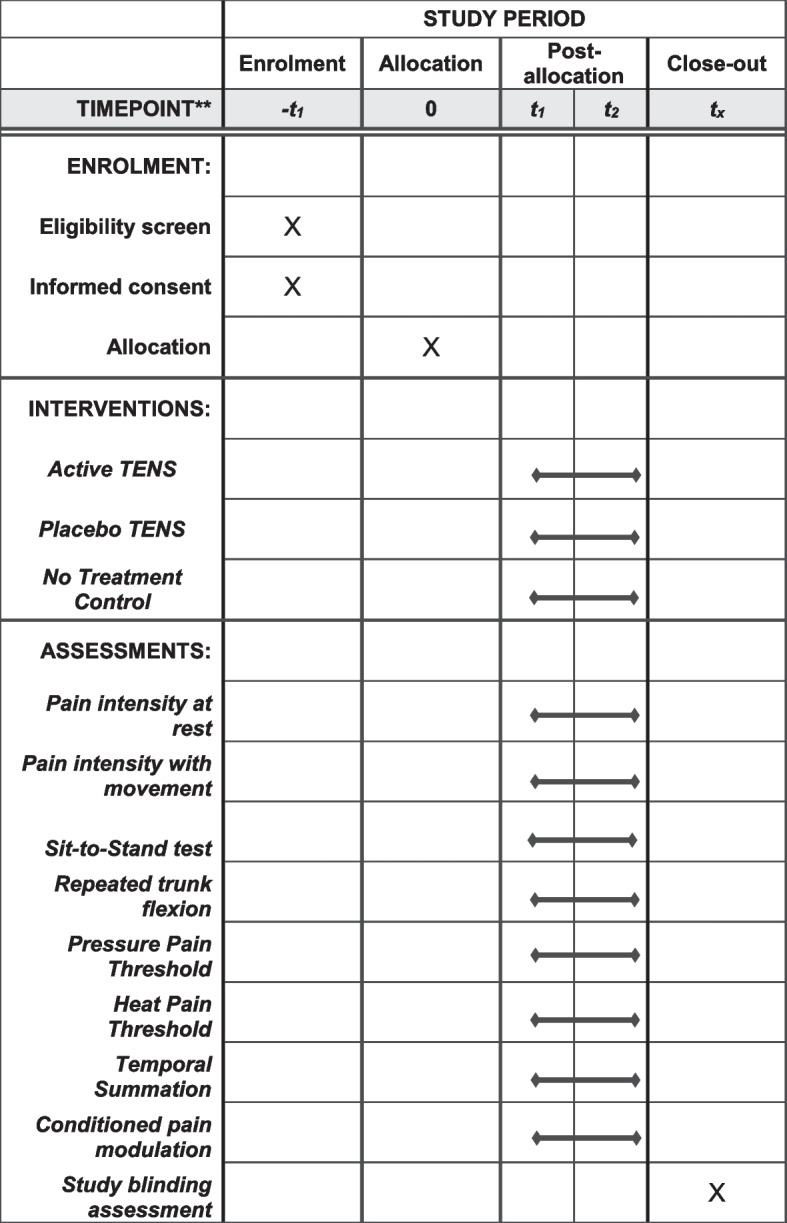
Schedule of enrolment, interventions, and assessments

An overview of the study procedures is shown in Fig. 1.

### Sample size {14}

Sample size was calculated to determine the total number of study participants needed to detect a difference of 2 points for the pain intensity outcome, which is considered a clinically relevant value for LBP [32], as measured using the NPRS, with a standard deviation of 1.9 points [13]. An 80% statistical power, 5% alpha, and a possible sample loss of 15% were considered. Thus, a total of 34 participants will be needed. This calculation was performed using Minitab v.17 software (State College, PA, USA).

### Recruitment {15}

Participants will be invited to participate in the study through social media sites (e.g., Facebook and Instagram), leaflets, paper and digital flyers, websites, and online news sources from the University of Hartford. After selecting the participants, a subjective interview will be conducted to assess the sample eligibility.

## Assignment of interventions: allocation

### Sequence generation {16a}

The order of active TENS, placebo TENS and no TENS will be randomized via the website (randomization.com).

### Concealment mechanism {16b}

Concealment of the treatment order will be achieved using sequential numbering in sealed opaque envelopes, which will be stored in a secure cabinet and only be opened before the first session by the researcher responsible for the application of the TENS treatments.

### Implementation {16c}

An independent researcher with no other role in this study will perform the randomization.

## Assignment of interventions: blinding

### Who will be blinded {17a}

Both the evaluator and data analysts will be blinded to the randomization and intervention processes. The participants and the researcher responsible for the treatment cannot be blinded due to the nature of the interventions.

### Procedure for unblinding if needed {17b}

The outcome evaluator and data analysts will not be allowed to unblock the blinding. However, the researcher responsible for applying the treatments will not be blinded.

## Data collection and management

### Plans for assessment and collection of outcomes {18a}

All researchers, including the person responsible for the interventions and the outcome evaluator, will undergo training before the experiment. Intra-examiner reliability will be estimated for the pressure pain threshold and temporal summation measurements by calculation of intraclass correlation coefficient (ICC). All data will be anonymized and stored in a folder on the institutional One Drive cloud. Only the study team will have access to this study folder.

### Plans to promote participant retention and complete follow-up {18b}

The researchers will be in weekly contact with the study participants. Twenty-four hours before treatment sessions, a researcher will send text messages to remind participants of the treatment date and time. This procedure will ensure that participants receive the necessary attention and will assist in fully accompanying them during the research. If any participant fails to attend the appointment, the researchers will immediately call to inquire about the reasons for the no-show. If participants abandon the trial, the reasons will be recorded, the most recent data will be compiled, and analysis will be performed using the intention-to-treat principle.

### Data management {19}

All the data will be collected weekly and stored on a secure computer server, with personal login access authorized by the principal investigator of the present study. All data collected in this trial will be restricted to the principal investigator and specific research team members.

### Confidentiality {27}

The information collected will remain anonymous; participants will be assigned a participant number for identification purposes. The data will be stored on the institutional One Drive of the University of Hartford which only the research team can access.

### Plans for collection, laboratory evaluation, and storage of biological specimens for genetic or molecular analysis in this trial/future use {33}

N/A. No biological specimens will be collected as part of this trial.

## Statistical methods

### Statistical methods for primary and secondary outcomes {20a}

An assessor blinded to the randomization and assessment processes will perform the data analysis for the primary and secondary outcomes. The intention-to-treat principle will be adopted in the analyses. Descriptive statistics (mean, standard deviation, 95% confidence intervals) will be generated for all variables. Normality will be evaluated using the Kolmogorov–Smirnov test and the Levene’s test will be used to assess for homogeneity of variance. Repeated measures ANOVA and the Tukey test will be used for normally distributed data. The Friedman test, followed by post hoc pairwise comparisons using the Wilcoxon Signed Ranks test, will be used if data are not normally distributed. Cohen’s d test will be used to calculate the effect size for normally distributed data and it was classified as small (0.0–0.2), moderate (0.3–0.5), or large (≥ 0.6) [33]. For non-normally distributed data the effect size will be calculated by the Cliff’s delta classified as small (0.147 ≤ 0.330), medium (0.330 ≤ 0.474), or large (≥ 0.474) [34]. These analyses will be performed using the SPSS v.28 statistical software (SPSS, Inc., IL, USA). Statistical significance will be considered at *p* <  0.05.

### Interim analyses {21b}

No interim analyses were planned.

### Methods for additional analyses (e.g., subgroup analyses) {20b}

Currently, there is no planned additional subgroup or adjusted analyses.

### Methods in analysis to handle protocol non-adherence and any statistical methods to handle missing data {20c}

The intention-to-treat analysis will be used, and the missing data will be handled appropriately following established guidelines [35].

### Plans to give access to the full protocol, participant-level data and statistical code {31c}

Any data required to support the protocol can be supplied on reasonable request to the corresponding author to promote study transparency. However, only de-identified datasets will be supplied.

## Oversight and monitoring

### Composition of the coordinating center and trial steering committee {5d}

The trial steering committee comprises three key members: one principal investigator and two co-investigators. This committee ensures the effective management of the trial. Duties encompass the approval of the final protocol and monitoring the ongoing progress of the study. The committee also has the authority to consider and agree upon modifications to the study protocol.

The trial management committee, comprising the two co-investigators, doctoral physical therapy students, and one independent primary investigator, is responsible for study planning and day-to-day management of the trial. The role of the group is to monitor all aspects of the conduct and progress of the trial, engage in the patient recruitment process, ensure that the protocol is adhered to, and take appropriate action to safeguard participants and the quality of the trial itself. The independent primary investigator will be responsible for generating the allocation sequences of the randomization. Meetings are held weekly.

### Composition of the data monitoring committee, its role and reporting structure {21a}

We will conduct the study without a Data Monitoring Committee. The principal investigator will organize and monitor the data obtained in this research project.

### Adverse event reporting and harms {22}

All participants will be carefully evaluated weekly, and all adverse events related to treatments will be reported, if any. The study related adverse events will be classified by the evaluator according to their severity as mild, moderate, or severe.

### Frequency and plans for auditing trial conduct {23}

The principal investigator will permit study-related audits, and inspections by the IRB and applicable granting agencies or regulatory bodies, including access to all study-related documents.

### Plans for communicating important protocol amendments to relevant parties (e.g., trial participants, ethical committees) {25}

Any changes to the protocol will be reported to the IRB of the University of Hartford for approval.

### Dissemination plans {31a}

This trial's results will be presented at scientific conferences and published in a peer-reviewed medical journal.

## Discussion

TENS is a non-pharmacological and non-invasive resource widely used by healthcare professionals and in clinical studies to reduce different types of pain. The hypoalgesic effects depend on the use of adequate stimulation parameters and researchers must assess pain during or immediately after TENS [36]. Moreover, pain should be assessed at rest and during movement. Unfortunately, previous studies assessing the effects of TENS in patients with chronic low back pain have failed to use proper intensities of current, and the assessment of pain was not performed during movement or during the peak of the analgesic response [13, 37].

In this trial, we will assess the immediate effects of TENS using the maximal tolerable intensity on measures of rest and movement-induced pain, function, and descending inhibition in participants with chronic non-specific back pain. We hypothesize that people suffering from chronic non-specific back pain can experience significant relief in both rest and movement-related back pain by receiving TENS at the highest intensity. This relief should occur immediately after TENS application, which distinguishes it from the outcomes of groups receiving a placebo or no treatment. Our protocol aims to go beyond pain management, as we believe that TENS can also improve function, as assessed by the sit-to-stand test and repeated trunk flexion. From a mechanistic perspective, we believe that TENS can enhance the descending inhibition by activating analgesic pathways.

## Trial status

The study protocol was approved by the IRB of the University of Hartford on March 27, 2023 (Prot. n. 23–03-187) and was registered at ClinicalTrials.gov on May 24, 2023, with registration number NCT05812885. Recruitment of participants will start in August 2023 and is expected to be completed in August 2024.

## Data Availability

The datasets used and/or analyzed will be available from the corresponding author upon reasonable request after the study is complete.

## Abbreviations

CPM: Conditioned pain modulation
CT: Connecticut
HPT: Heat pain threshold
ICC: Intraclass correlation coefficient
IRB: Institutional Review Board
LBP: Low back pain
NPRS: Numerical Pain Rating Scale
PPT: Pressure pain threshold
TS: Temporal summation
TENS: Transcutaneous electrical nerve stimulation
